# Suspension of Esophageal Stent: An Endoscopic Technique for the Treatment of Esophagopleural Fistula

**DOI:** 10.5152/tjg.2024.23461

**Published:** 2024-05-01

**Authors:** Chunsi Zheng, Jingbo Yang

**Affiliations:** 1 Shantou University Medical College, Shantou, China; 2 Department of Gastroenterology, Shenzhen Second People’s Hospital/The First Affiliated Hospital of Shenzhen University, Shenzhen, China

## Case Presentation

Endoscopic stenting is one of the most effective therapy options for gastrointestinal transmural defects, while the high stent migration rate is still noteworthy.^1,2^ Here we report a case of successful anchor and suspension technique with dental floss, an endoscopic technique for esophageal stent fixation. A 58-year-old man had undergone laparoscopic sigmoid colectomy and chemotherapy for sigmoid adenocarcinoma 4 months earlier. He complained of chest pain and dyspnea for 12 hours. Chest computed tomography identified a right pneumothorax, a right pleural effusion, mediastinal air, and esophageal wall disruption (Figure 1). A chest tube was placed to drain the pleural effusion. Gastroduodenoscopy (GIF-Q260J; Olympus, Tokyo, Japan) revealed a fistula 30 cm from the incisors, with a 0.7 cm longitudinal diameter, between the esophagus and pleural cavity. Based on the above findings, an esophagopleural fistula was suspected.

## Technique

Grasping forceps (JHY-FG-23-160-A1, Jiuhong Medical, Changzhou, China) was used to scratch the mucosa around the fistula orifice to initiate and promote inflammation and thus tissue regeneration for fistula closure. A fully covered self-expanding metal stent (Bonastent, BE-2014 20mm; Sewoon Medical, Chungcheongnam-do, Korea) was placed along the esophagus to cover the fistula orifice. Dental floss (Oral-B Glide Floss) was strong, shred-resistant, and able to withstand longitudinal traction, thus being chosen for stent fixation. The dental floss was knotted into a circle of approximately 2 cm in diameter, similar to the diameter of the stent. Then the circle was grasped with a metal clip (Eco Clip, ROCC-D-26-195 11mm; Micro-Tech, Nanjing, China) and advanced to the esophagus. About 5-6 clips were needed to anchor the dental floss circle around the proximal flange of the stent to maintain the balance of the traction force in different directions (Figure 2). Regular pentagonal or hexagonal shapes of the circle were not rigidly required. The technical pointer was achieved when the dental floss was tightened under endoscopic observation. The dental floss was then drawn out of the nose and threaded into a sputum suction catheter to minimize nasal and oropharyngeal irritation. The end of the sputum suction catheter was covered with gauze and held in place below the ear with tape to prevent stent migration (Figure 3). The patient was kept on nasojejunal feeding after the operation and finally transitioned to oral feeding. The stent retention was confirmed with a chest x-ray after 3 months (Video 1). After removing the stent, gastroduodenoscopy showed a fully recovered esophagopleural fistula (Figure 4).

## Conclusion

Different endoscopic techniques, including through-the-scope clips, over-the-scope clips, stentfix OTSC, and endoscopic suturing, have been investigated to lower the rate of esophageal stent migration. The overall technical success rates are 96.7%-98%, the clinical success rates are 72%-79%, and the migration rates range from 5.1% to 17.2%.^2-4^ Despite the comparatively high success rate and low stent migration rate, there are some limitations, such as device availability, technical difficulty, and procedural costs. Endoscopic suturing requires more specific technique training; the tangential working angle makes adequate suture depth challenging.^3^ Specific cutting instruments are generally required for OTSC fragmentation and retrieval.^5^ Some novel and advanced endoscopic instruments may not be available in some institutions, especially those in remote areas. The technique introduced in this case is a simple external fixation method that is not limited to the type and size of stent and is easy to perform by endoscopists, which may be capable of being executed in medical institutions. Nonetheless, endoscopists should inform the patients about nasopharyngeal discomfort and get their cooperation. Besides technological complexity and clinical success rate, it would be preferable to take the patient’s general health and financial status into account when making healthcare decisions.

^*^The video file linked to this article is available in the online version of the journal. Or you can utilize the QR code provided on this page to gain access.


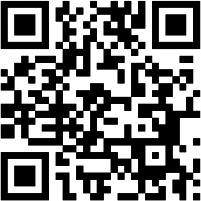


## Figures and Tables

**Figure 1.**
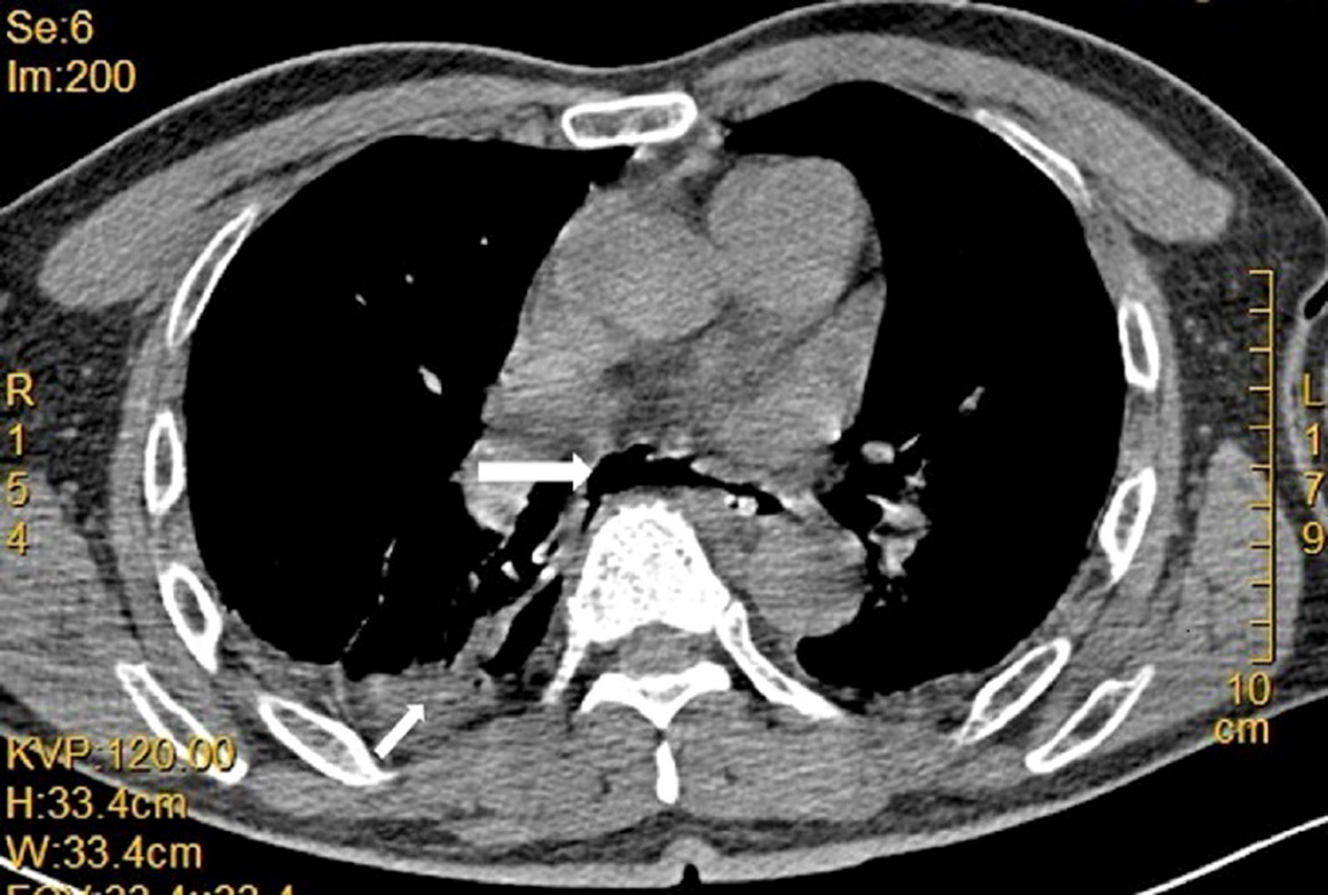
Computed tomography scan identified right-sided hydropneumothorax and pneumomediastinum.

**Figure 2.**
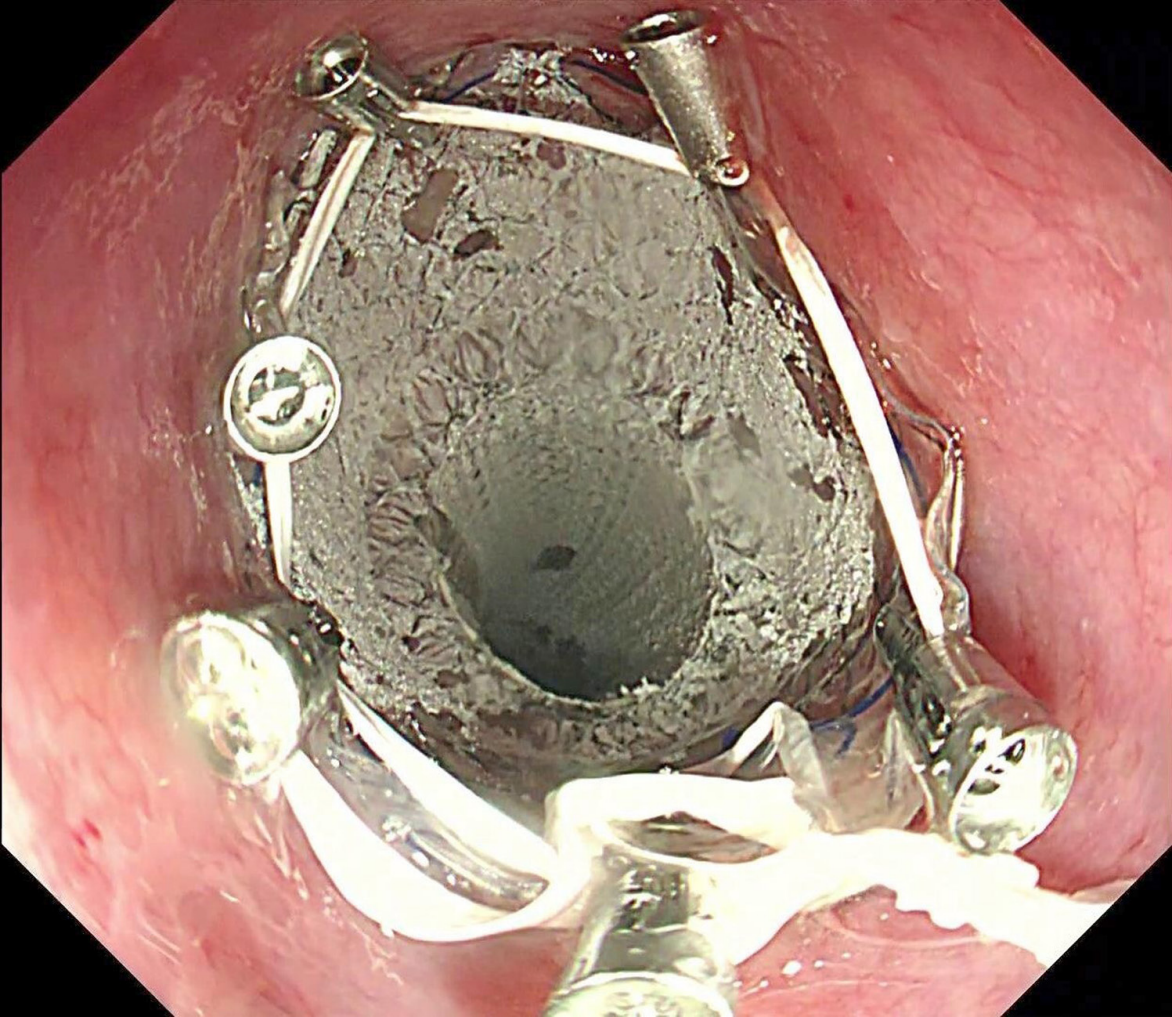
Anchoring the proximal flange of the stent with six metal clips connected with dental floss.

**Figure 3.**
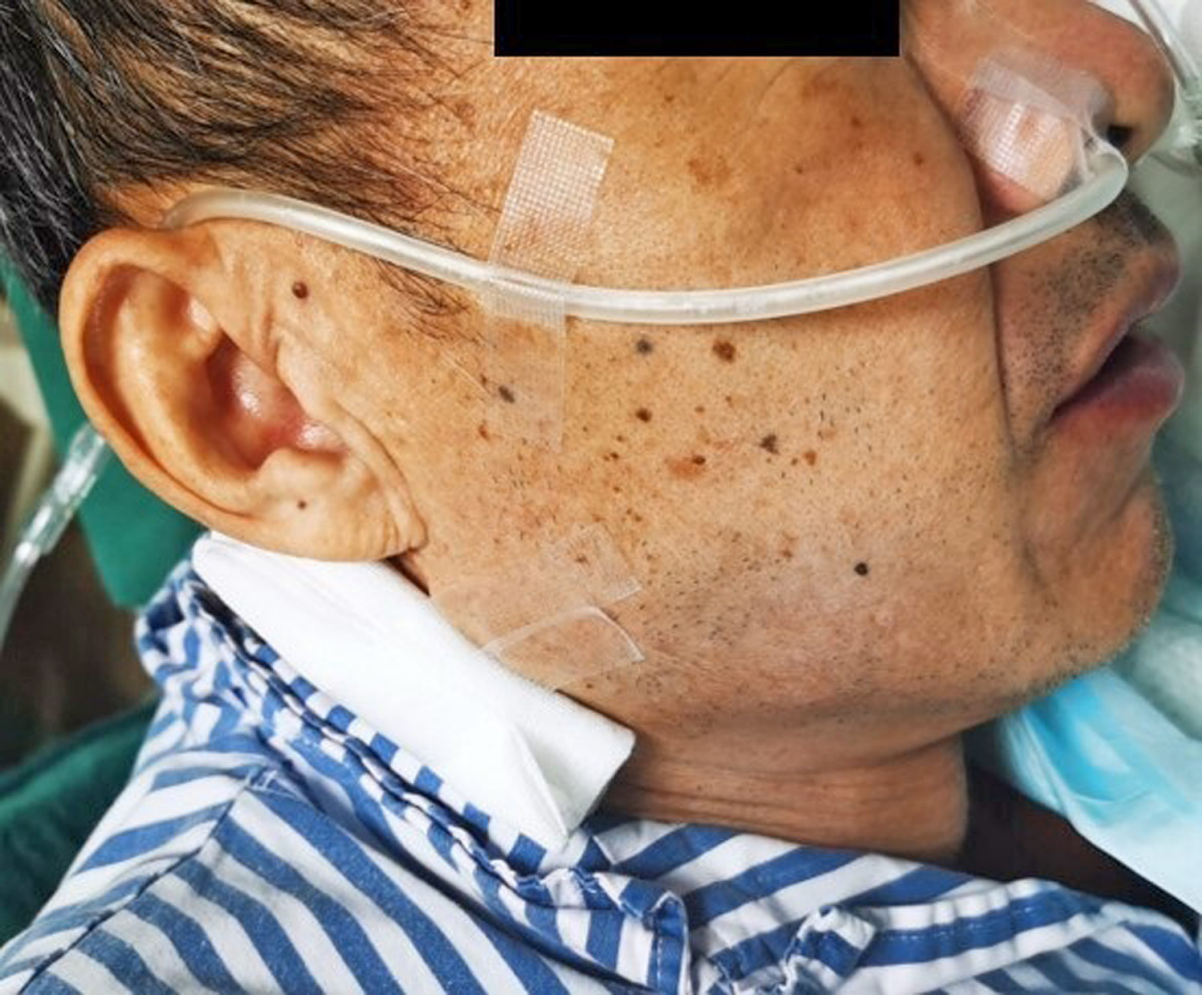
The dental floss was then drawn out of the nose, threaded into a sputum suction catheter, and attached to the earlobe to prevent stent migration.

**Figure 4.**
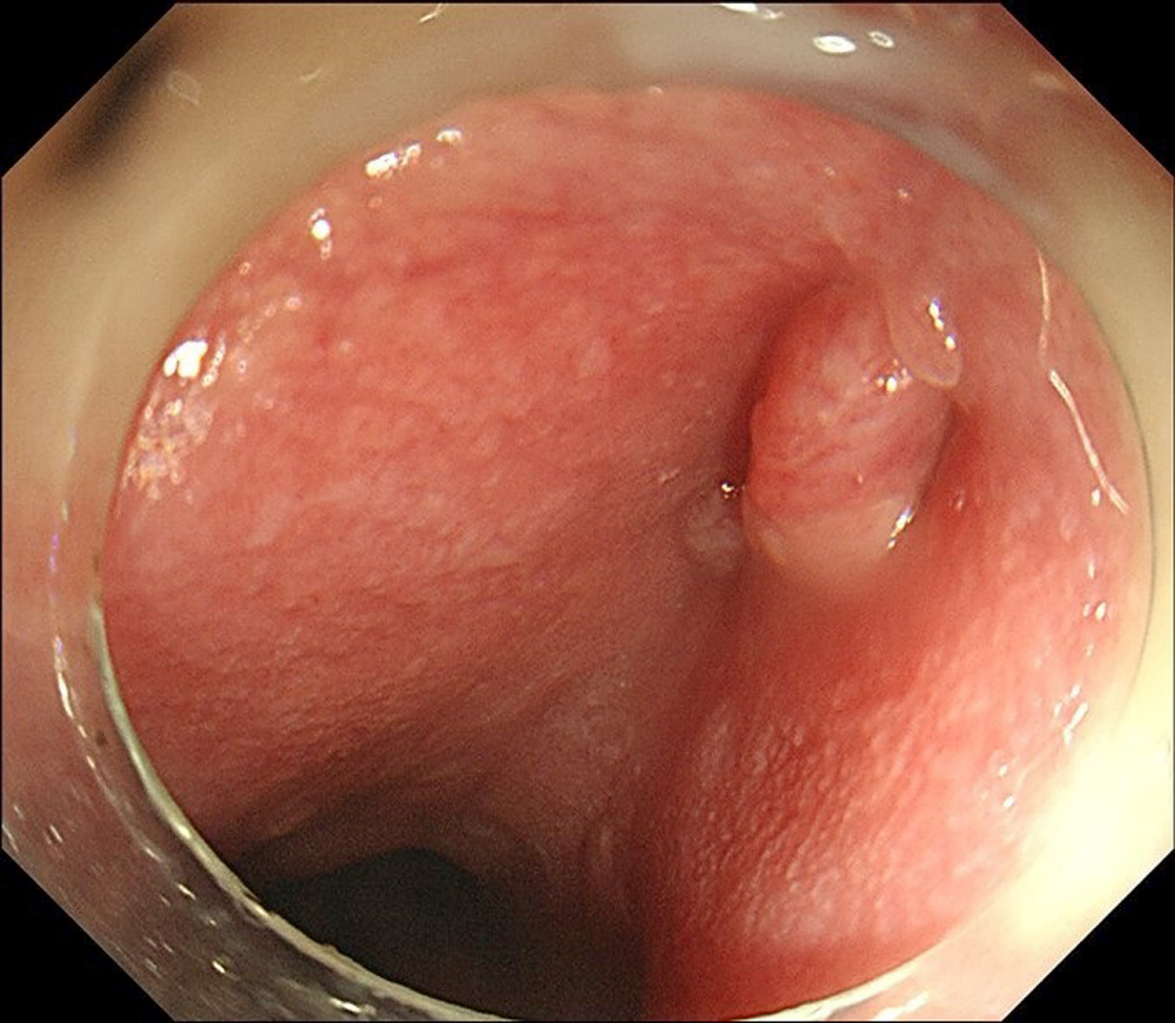
Gastroduodenoscopy showed a fully recovered esophagopleural fistula.
